# Deep autoencoder for low dimensionality for high dimensional data in regression models and direct inverse analysis of models

**DOI:** 10.1007/s44211-026-00904-2

**Published:** 2026-04-06

**Authors:** Hiromasa Kaneko

**Affiliations:** Department of Applied Chemistry, School of Science and Technology, Meiji University, 1-1-1 Higashi-Mita, Tama-ku, Kawasaki, Kanagawa 214-8571 Japan

**Keywords:** Machine learning, High dimension, Deep autoencoder, Spectra, Profile, Time-series

## Abstract

In constructing machine learning models between features X and output variables Y in molecular, material, and process designs, and process control, when the number of X is huge, models are likely to overfit, and it takes time to construct models. Deep autoencoder (DAE) was focused on, and reduction of dimensions in the huge number X with DAE was investigated. Spectral data, profile data, and time-series data were used to virtually generate data whose number of X was 10,000, 100,000, and 1,000,000. For each dataset, DAE model was constructed with training data, and then test data of X was reconstructed by transforming X into latent variables Z and Z into X, and then, it was confirmed that DAE could reconstruct X with high reproducibility regardless of the number of X in all the datasets. In addition, regression models were constructed with Gaussian mixture regression (GMR), which could perform direct inverse analysis of model, between Z and Y, and then, Y could be predicted accurately regardless of the number of X. It was confirmed that DAE could appropriately reduce the dimension even when X was as large as 1 million. Furthermore, by combining DAE with GMR, it became possible to directly predict X values from target Y values by calculating Z from Y with GMR and transforming Z into X with DAE.

**Graphical abstract:**

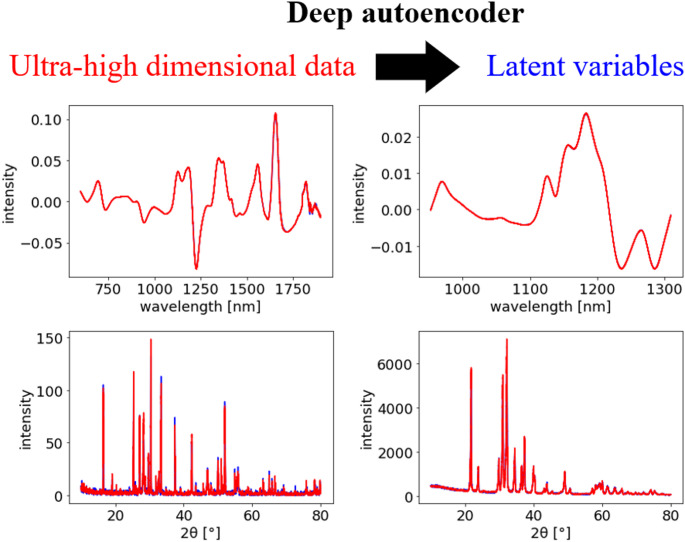

## Introduction

In molecular, material, and process designs, and process control, a mathematical model Y = f(X) is constructed between features X such as molecular descriptors, experimental conditions, synthesis conditions, manufacturing conditions, evaluation conditions, process conditions, and process variables, and output variables Y such as physical properties, activities, and characteristics of molecules and materials and product quality with machine learning. Y values can be predicted by inputting X values into the constructed model, and X values for which Y is the target value can be designed.

Incorporating detailed information on molecules, materials, and processes into X is expected to improve the prediction accuracy of the model. Candidates of X for molecules and materials include analytical data such as spectroscopic spectra and X-ray diffraction (XRD). Neo et al. [1] reviewed machine learning with near-infrared spectroscopy, mid-infrared spectroscopy, Raman spectroscopy and laser-induced breakdown spectroscopy as X for sorting plastic waste in the recycling industry. Ellis et al. [2] used Fourier transform infrared (FT-IR) spectroscopy as X of machine learning model to estimate the microbial loads of food samples. Kaneko et al. [3] used near-infrared spectroscopy as X of machine learning model to predict glucose and lactate concentrations in culture media. Zhu et al. [4] quantified species of volatile organic compounds and their concentrations from the wavelength and intensity of spectra signals with the enhancement from plasma using machine learning model. Lansford and Vlachos [5] combined machine learning and infrared spectra associated with activating adsorbate vibrational modes for characterizing surface microstructure of complex materials.

Surdu and Győrgy [6] reviewed XRD data analysis by machine learning including XRD classification and phase identification, lattice and quantitative phase analyses, and detection of defects and substituents, as well as microstructural material characterization. Ali et al. [7] reviewed XRD techniques and application of machine learning for mineral characterization. Kersch et al. used Raman and infrared spectra and XRD profiles for phase identification in doped HfO_2_ and ZrO_2_ [8]. Kireeva et al. [9] used machine learning methods to obtain the models predicting the electrochemical and structural characteristics of Li-rich layered oxide cathode materials, and the data from XPS spectra and XRD profiles were used as the additional X to evaluate the target functional characteristics: initial discharge capacity, coulombic efficiency and capacity fade. Cao et al. [10] predicted compressive strength of portland cement clinker using machine learning and XRD profiles. Motojima et al. [11] constructed machine learning models predicting bone formation rate from Fourier transform infrared spectra and XRD profiles of bone substitute materials, and direct inverse analysis of the models was conducted.

The more advanced the analysis, and thus the more detailed information is included in X, the larger the number of X. In process designs and control, when X includes time series data, the number of X increases with the amount of time to be considered. The more complex the equipment in the process, the greater the number of process variables, and (number of process variables) × (number of time) becomes the number of X, which is huge.

When the number of X is large, not only is the model likely to overtfit due to chance correlations, but it also takes more computation time in model building. For example, in linear regression analysis, it is necessary to optimize as many parameters as the number of X. In decision trees and random forests, the number of parameters to be optimized increases and the computational complexity increases, as the number of X increases. In Gaussian mixture regression (GMR) [12, 13], because the variance-covariance matrix must be calculated between X and Y, the computational complexity increases exponentially with the number of X.

Kaneko previously confirmed that for datasets such as molecular, material, and spectral datasets, using a deep autoencoder (DAE) to convert X to a latent variable Z with dimensionality reduction and regression analysis between Z and Y suppresses model overfitting and improves the prediction accuracy of regression models compared to regression analysis between X and Y [14]. The model constructed with GMR, which was used as the regression analysis method, allowed direct inverse analysis of model. Z values can be predicted directly from the target value of Y. Then, because the decoder in DAE can transform Z to X, it is possible to directly predict Z from Y using GMR, and X from Z using DAE. The direct inverse analysis of machine learning model can be achieved by combining GMR and DAE.

Latent variable transformation is conducted with DAE for data sets with high X, such as spectral data, XRD profiles, and time series data with as many as 1 million X, GMR models are constructed with latent variables and Y, and then, direct inverse analysis of the constructed models is performed and 1 million X is predicted with high reproducibility.

## Method

The basic autoencoder is a neural network with three layers, namely an input layer, a hidden layer, and an output layer, whereas a neural network with multiple hidden layers is denoted as a deep autoencoder. When **x** is given, the following equation is used to encode **x** to the values of the first hidden layer **z**_1_ ϵ R^*p*1^ (where *p*_1_ is the number of neurons) in the encoder.

1 $${{\mathbf{z}}_1}=g\left( {{{\mathbf{W}}_1}{\mathbf{x}}+{b_1}} \right),$$

where **W**_1_ and *b*_1_ are weights and bias of the encoder, respectively, and *g* is an activation function. Herein, the ReLU function [15] was used as the activation function. The transformation from the first hidden layer to the second hidden layer, that from the second hidden layer to the third hidden layer, etc. were encoded as in Eq. (1). The following equation was used to decode the values of the first hidden layer **z**’_1_ ϵ R^*q*1^ (where *q*_1_ is the number of neurons) to the reconstructed sample **x**’ at the decoder.

2 $${\mathbf{x}}'=g\left( {{{\mathbf{G}}_1}{\mathbf{z}}{'_1}+{c_1}} \right),$$

where **G**_1_ and *c*_1_ are weights and bias in the decoder, respectively. The transformation from the first hidden layer to the second hidden layer, that from the second hidden layer to the third hidden layer, etc., were decoded as in Eq. (2). Herein, the deep autoencoder was trained to minimize the mean absolute error between **x** and **x′**, and Adam [16] was used as the optimizer. The DAE calculation used Keras 2.0.5 [17].

In this paper, the term “network structure” refers to the numbers of neurons in each hidden layer of DAE, written as a comma-separated sequence from the encoder side to the decoder side. The smallest number in the sequence corresponds to the bottleneck layer and therefore to the dimensionality of latent variables z. For example, “32, 16, 8, 16, 32” denotes a symmetric DAE with an encoder 32→16→8 (bottleneck) and a decoder 8→16→32. Table 1 lists the candidate structures examined in this study; the final structure was selected from these candidates based on reconstruction performance on the training/validation data.

**Table 1:** Network structure candidates for DAE

8
16
32
16, 8, 16
32, 16, 32
64, 32, 64
32, 16, 8, 16, 32
64, 32, 16, 32, 64
128, 64, 32, 64, 128
64, 32, 16, 8, 16, 32, 64
128, 64, 32, 16, 32, 64, 128
256, 128, 64, 32, 64, 128, 256

This study combines DAE with supervised learning. Generally, a model Y = f(X) is constructed by supervised learning between X and Y. When Y is quantitative and continuous, it is a regression analysis; when Y is qualitative information, it is a classification. When X is high dimensional, not only it is easy to overfitting due to chance correlation, but it also takes an enormous amount of time to construct the model. For example, in GMR, because the model is represented by a superposition of Gaussian distributions among all variables including X and Y, it is necessary to fit the parameters of (number of Gaussian distributions) × (number of X + number of Y) as the mean vector and the parameters of (number of Gaussian distributions) × (number of X + number of Y)^2^ as the variance-covariance matrix. Because the number of parameters increases exponentially as the number of X increases, model construction is not realistic in terms of computation time when X is high dimensional.

After transforming X to Z with DAE, a machine learning model Y = f(Z) is constructed between Z and Y. Even when X is high dimensional, by reducing the dimension using DAE, regression and classification models can be constructed in a short time without overfitting the model. Even in GMR, the above (number of X) becomes (number of Z), the mean vector and variance-covariance matrix can be fitted in a realistic amount of time.

GMR can not only predict Y from Z, but also predict Z from Y, which is impossible for the other general regression analysis methods. Because DAE can not only transform X to Z, but also inverse transform Z to X, combining DAE and GMR can achieve Z to X prediction by DAE after Y to Z prediction by GMR, making it possible to predict X from Y directly, which means direct inverse analysis of machine learning model. GMR is available at DCEKit [18].

## Results and discussion

To verify the performance of DAE and the predictive ability of the combination of DAE and GMR for high dimensional X, the tablet datasets of Shootout2002 [19] (API1) and Shootout2012 [20] (API2) as spectral datasets, XRD datasets [21], and time-series datasets were used. The Savitzky–Golay method [22] was used to preprocess the spectra. The window size was 21, the polynomial was of second order, and first-order derivatives were used. The XRD datasets included rapid and slow scans (XRD rapid and XRD slow) over a range of 2θ = [10°, 80°]. The time-series data (Time-series) were generated with T-Gen [23] based on a sulphur recovery unit dataset [24]. The numbers of samples and X are shown in Table 2. In the tablet datasets (API1 and API2), Y represents the reference assay value (content/concentration) of the active pharmaceutical ingredient provided with the Shootout datasets. The XRD and time-series datasets do not include Y and are therefore used only for evaluating reconstruction performance of DAE.

The data were randomly split so that the training and test sets contained 70% and 30% of the samples, respectively. The network structure of the deep autoencoder was selected from Table 1. Five-fold cross-validation was performed to determine the hyperparameters for GMR.

**Table 2:** The number of samples for each dataset

API1	# of samples	# of X
	655	650
API2	228	372
XRD rapid	300	1763
XRD slow	220	6442
Time-series	50	10,081

**Fig. 1:**
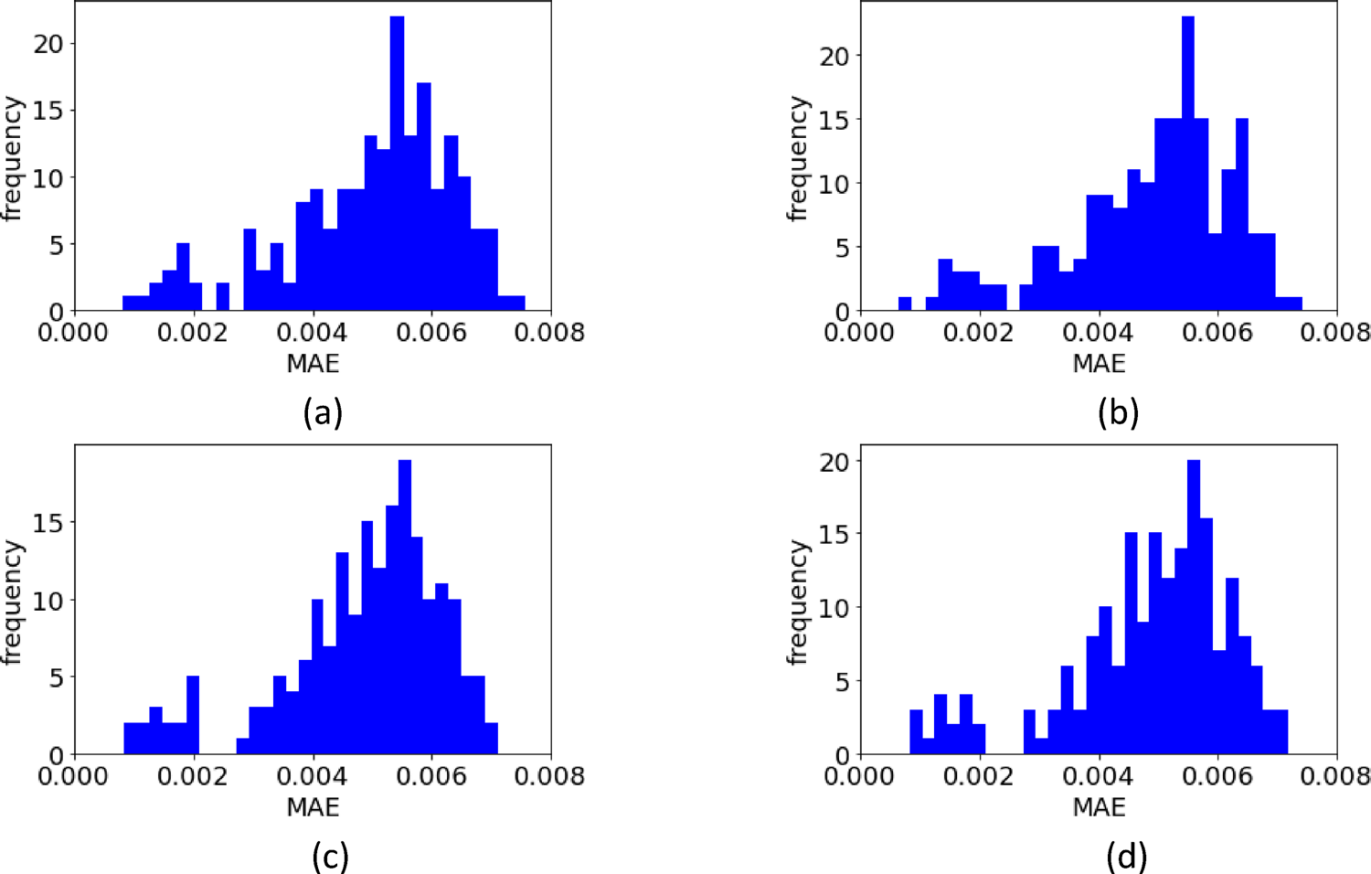
Histogram of MAE per sample of X using test data for each number of X in API1. **a** # of X: 655, (raw), **b** # of X: 10,000, **c** # of X: 100,000, **d** # of X: 1,000,000

**Fig. 2:**
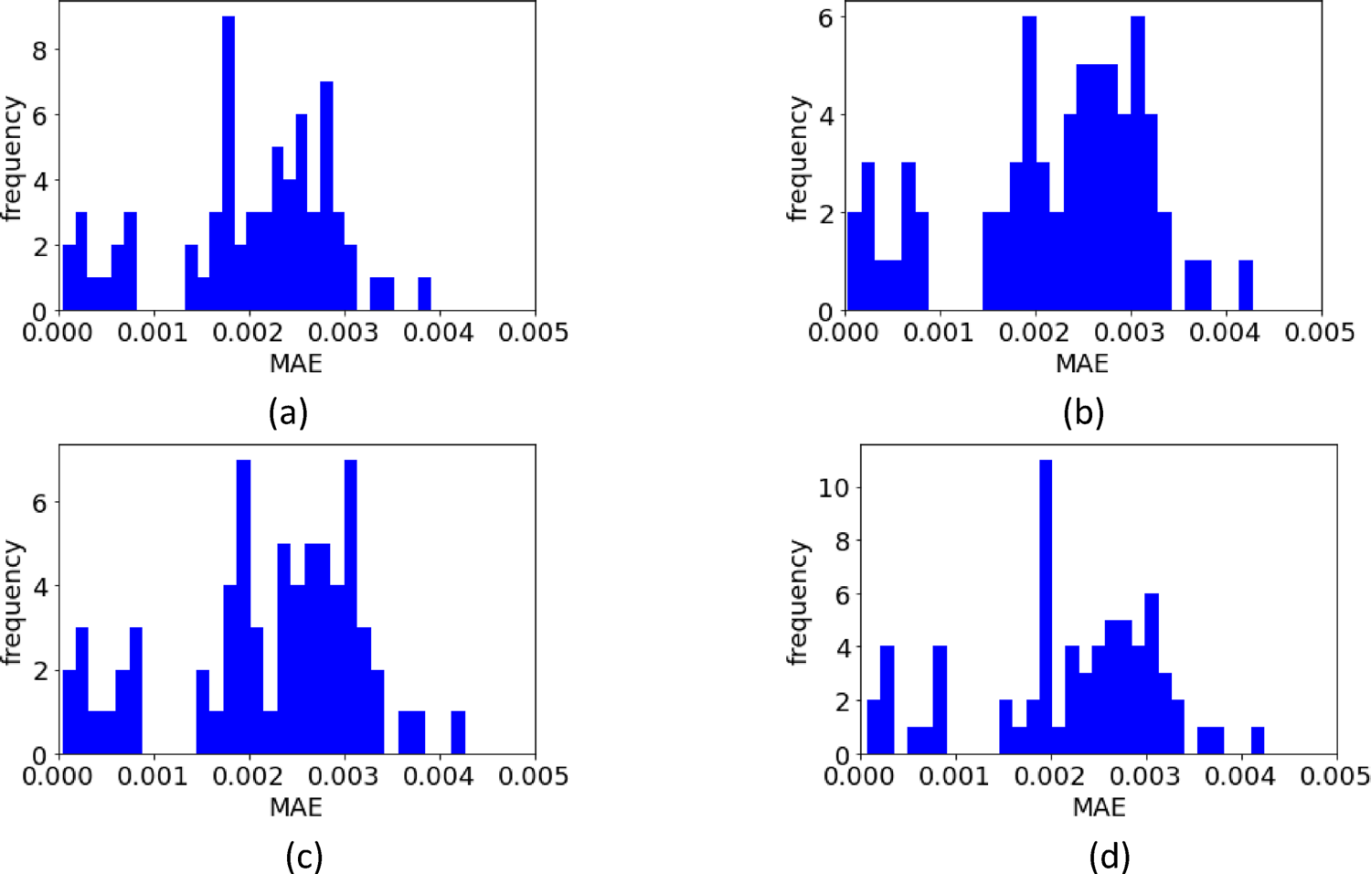
Histogram of MAE per sample of X using test data for each number of X in API2. **a** # of X: 372, (raw), **b** # of X: 10,000, **c** # of X: 100,000, **d** # of X: 1,000,000

**Fig. 3:**
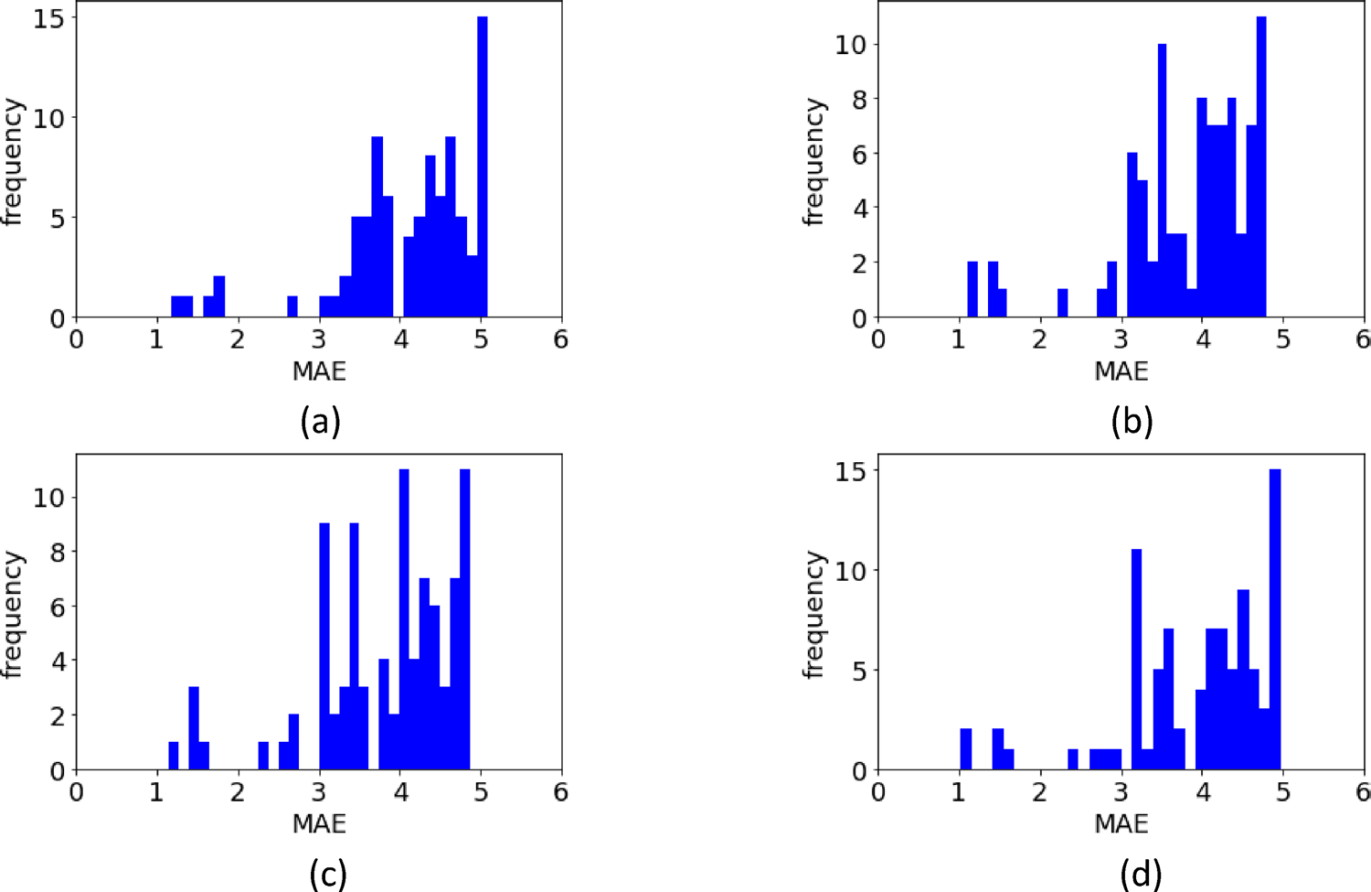
Histogram of MAE per sample of X using test data for each number of X in XRD rapid. **a** # of X: 1763, (raw), **b** # of X: 10,000, **c** # of X: 100,000, **d** # of X: 1,000,000

**Fig. 4:**
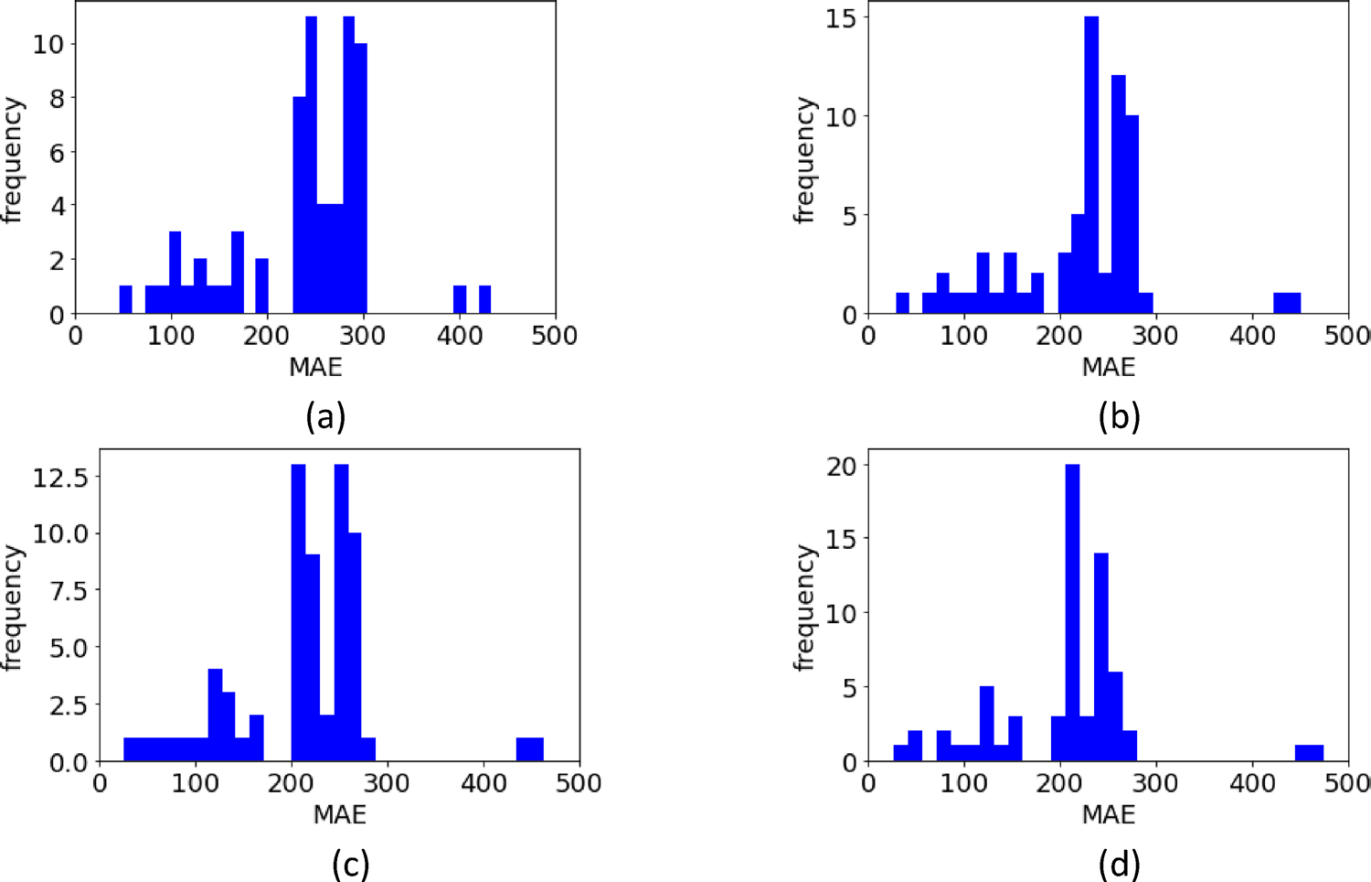
Histogram of MAE per sample of X using test data for each number of X in XRD slow. **a** # of X: 6442, (raw), **b** # of X: 10,000, **c** # of X: 100,000, **d** # of X: 1,000,000

**Fig. 5:**
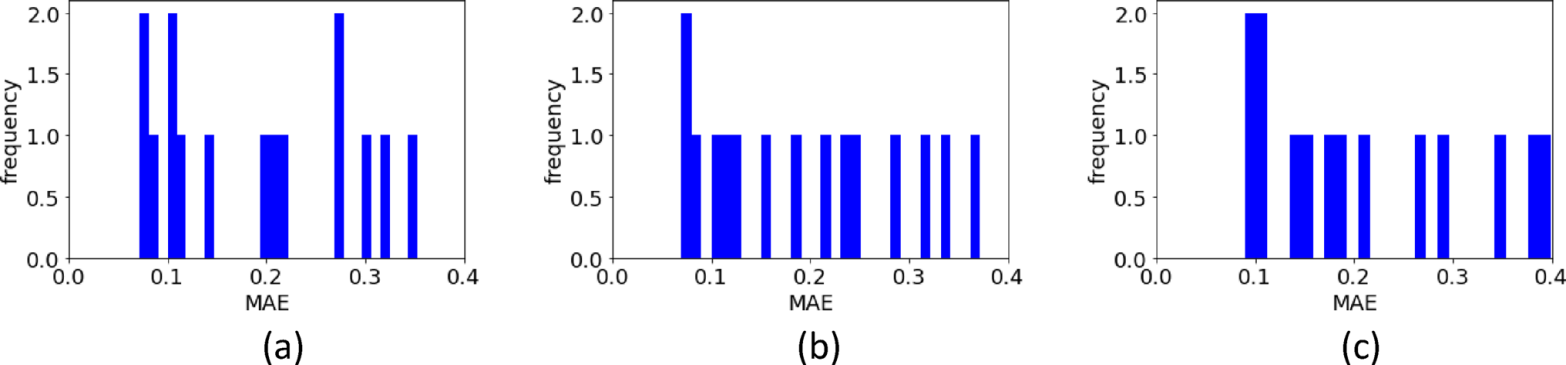
Histogram of MAE per sample of X using test data for each number of X in Time-series. **a** # of X: 10,081, (raw), **b** # of X: 100,000, **c** # of X: 1,000,000

For each of API1, API2, XRD rapid, and XRD slow, virtual samples whose numbers of X were 10,000, 100,000, and 1,000,000 were generated using linear interpolation with the original X values of each sample. For time-series, because the number of X or the number of time points in the original data set was 10,081, virtual samples whose numbers of X were 100,000 and 1,000,000 were generated. For each dataset, DAE model was trained using training data, and then, X of test data was reconstructed with the constructed DAE model by transforming X into Z using the encoder of the model and transforming Z into X using the decoder of the model. Then, mean absolute error (MAE) was calculated between original X and reconstructed X for each sample. For each number of X, the histograms of MAE in test data of API1, API2, XRD rapid, XRD slow, and Time-series are shown in Figs. 1, 2, 3, 4 and 5, respectively. Compared to the distribution of MAE for the raw X, we observed no substantial difference in the MAE distribution when the number of X was increased for each dataset. Here, “no substantial difference” is intended as a descriptive statement based on comparing the shapes of the MAE-per-sample histograms across different numbers of X; no formal hypothesis testing was conducted because this analysis aims to confirm that reconstruction quality does not systematically deteriorate as the dimensionality of X is increased. It was confirmed that DAE could appropriately reduce the dimensionality even when the number of X was as large as 1 million.

Figures 8, 9, 10, 11 and 12 in Appendix show examples of comparing the original X with the reconstructed X in the test data of API1, API2, XRD rapid, XRD slow, and Time-series, respectively. The fact that the red and blue lines overlap, and the blue line is not visible means that the DAE could reconstruct X with almost no error for test data. It was confirmed that the DAE could reconstruct X appropriately even when X was a huge number like 1 million.

**Fig. 6:**
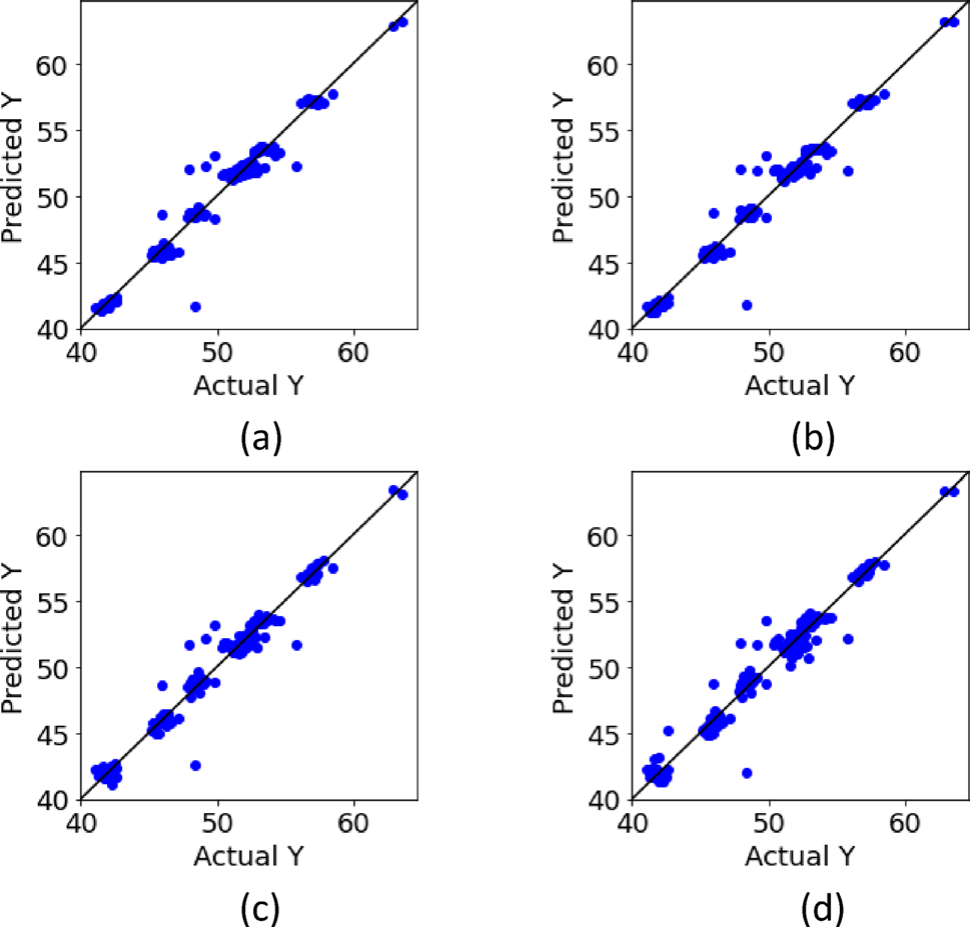
Actual Y versus predicted Y using test data for each number of X in API1. **a** # of X: 655, (raw), **b** # of X: 10,000, **c** # of X: 100,000, **d** # of X: 1,000,000

**Fig. 7:**
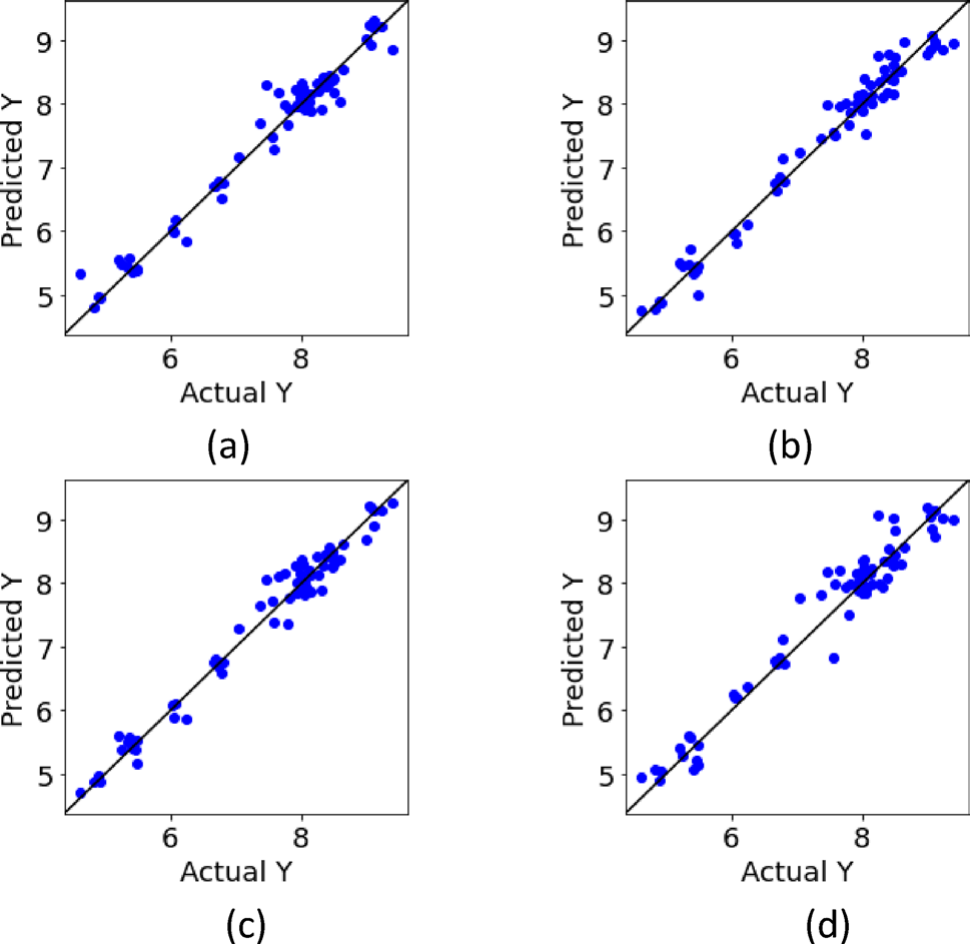
Actual Y versus predicted Y using test data for each number of X in API2. **a** # of X: 372, (raw), **b** # of X: 10,000, **c** # of X: 100,000, **d** # of X: 1,000,000

**Fig. 8:**
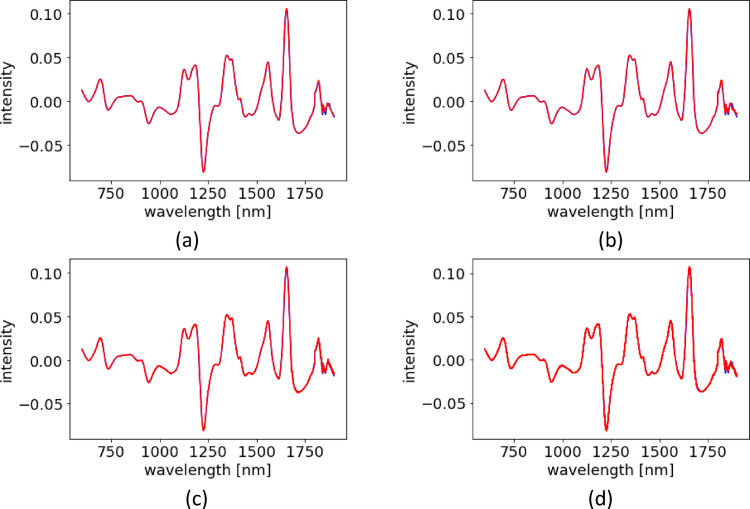
Example of the deep autoencoder (DAE) results for each number of X in API1. Blue line means an original X and red line means X reconstructed with DAE. The blue lines are difficult to see because the red lines are superimposed on the blue lines. **a** # of X: 655, (raw), **b** # of X: 10,000, **c** # of X: 100,000, **d** # of X: 1,000,000

**Fig. 9:**
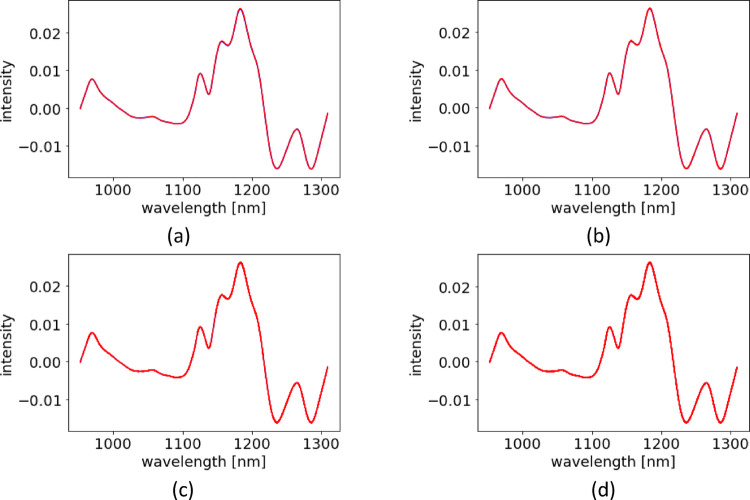
Example of the deep autoencoder (DAE) results for each number of X in API2. Blue line means an original X and red line means X reconstructed with DAE. The blue lines are difficult to see because the red lines are superimposed on the blue lines. **a** # of X: 372, (raw), **b** # of X: 10,000, **c** # of X: 100,000, **d** # of X: 1,000,000

**Fig. 10:**
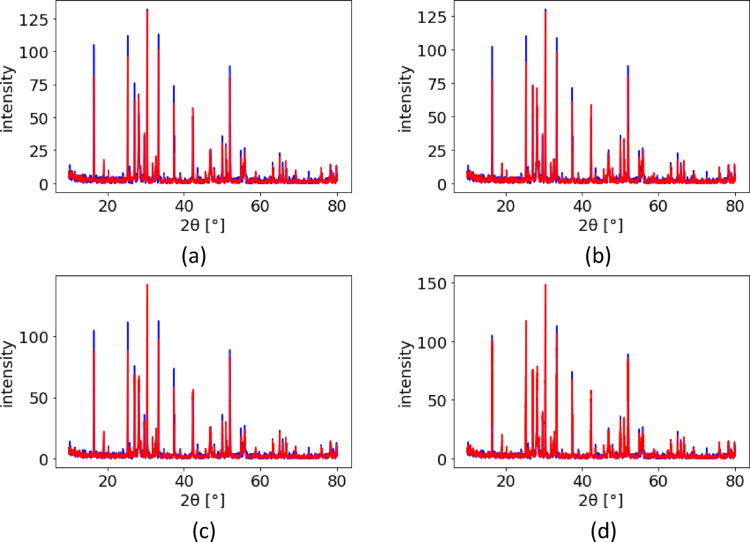
Example of the deep autoencoder (DAE) results for each number of X in XRD rapid. Blue line means an original X and red line means X reconstructed with DAE. The blue lines are difficult to see because the red lines are superimposed on the blue lines. **a** # of X: 1763, (raw), **b** # of X: 10,000, **c** # of X: 100,000, **d** # of X: 1,000,000

**Fig. 11:**
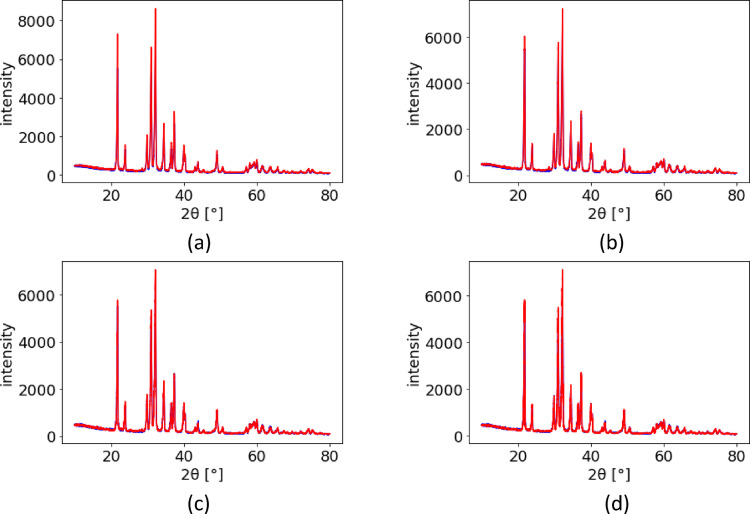
Example of the deep autoencoder (DAE) results for each number of X in XRD slow. Blue line means an original X and red line means X reconstructed with DAE. The blue lines are difficult to see because the red lines are superimposed on the blue lines. **a** # of X: 6442, (raw), **b** # of X: 10,000, **c** # of X: 100,000, **d** # of X: 1,000,000

**Fig. 12:**
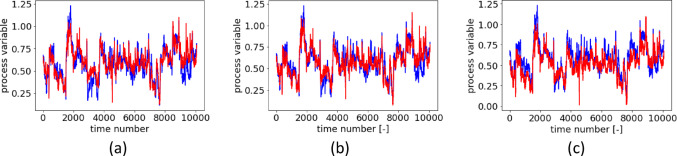
Example of the deep autoencoder (DAE) results for each number of X in Time-series. Blue line means an original X and red line means X reconstructed with DAE. **a** # of X: 10,081, (raw), **b** # of X: 100,000, **c** # of X: 1,000,000

As Y existed for API1 and API2, the construction of regression models between Z and Y using GMR after converting to Z using DAE, and the prediction using the constructed models were investigated. The results of predicting test data using the GMR model constructed with training data are shown in Tables 3 and 4, and Figs. 6 and 7. R^2^_TEST_ is the determinant coefficient of test data and RMSE_TEST_ is root-mean-squared error of test data. For both API1 and API2, there was almost no changes in R^2^_TEST_ and RMSE even when the number of X was increased, and there was also no substantial difference in the actual Y vs. predicted Y plots. Even when the number of X values was as large as 1 million, it was possible to construct a model with high prediction accuracy using GMR by reducing the dimensionality using DAE. In addition, because the GMR model can perform direct inverse analysis, i.e. it can predict Z from Y, and it can convert Z to X using DAE, by inputting the desired Y value into the GMR model, it is possible to calculate Z, and then calculate X from that Z using DAE, and therefore, it is possible to directly obtain the X value from the Y value.

**Table 3:** Comparison of predictive ability using test data for each number of X in API1

# of X	*R* ^2^_TEST_	RMSE_TEST_
650 (raw)	0.965	0.836
10,000	0.965	0.839
100,000	0.966	0.832
1,000,000	0.959	0.915

**Table 4:** Comparison of predictive ability using test data for each number of X in API2

# of X	*R* ^2^_TEST_	RMSE_TEST_
372 (raw)	0.966	0.237
10,000	0.972	0.216
100,000	0.974	0.207
1,000,000	0.950	0.293

## Conclusions

The reduction of features X into latent variables Z for data sets that can have a huge number of X, such as spectral data, profile data, and time series data, was investigated with deep autoencoder (DAE). The number of X was virtually changed to 10,000, 100,000, and 1,000,000, and each of the spectra, X-ray diffraction (XRD) profiles, and time series data were transformed from X to Z using the encoder of DAE, and then, Z was transformed to X using the decoder of DAE. It was confirmed that X could be reproduced accurately regardless of the number of X. Furthermore, for each data set with an objective variable Y, Gaussian mixture regression (GMR) model was constructed between Z and Y with training data and the prediction accuracy of the model was verified with test data, and as results, the prediction accuracy of the GMR model did not change much regardless of the number of X. It was confirmed that DAE could appropriately reduce the dimension even when the number of X is as large as 1 million.

The GMR model can be used for direct inverse analysis of the model, that is, direct prediction from Y to Z is possible, and thus, by combining GMR with DAE, it becomes possible to directly obtain X values that achieves a target Y value by predicting Z from Y using the GMR model and transforming Z to X using the DAE model.

Data was generated virtually when increasing the number of X to 10,000, 100,000, and 1,000,000, however, in the future, it will be necessary to verify the performance of DAE using actual experimental and analytical data sets, such as the results of high-resolution analysis. It will also be necessary to consider the dimensionality reduction using DAE with actual data that includes various variations and process states in time series data.

## Data Availability

The authors confirm that the data supporting the findings of this study are available within the article.

## Appendix

See Figs. 8, 9, 10, 11 and 12.
